# Occurrence of Ochratoxin A in Edible Pig Tissues (Kidneys, Liver, Muscle and Fat) in Greece Determined by HPLC-FD

**DOI:** 10.3390/toxins18040181

**Published:** 2026-04-08

**Authors:** Mikela Vlachou, Nikolaos Solomakos, Alexander Govaris, Vassilis Athanasiadis, Stavros I. Lalas, Andreana Pexara

**Affiliations:** 1 Laboratory of Hygiene of Foods of Animal Origin, Faculty of Veterinary Medicine, University of Thessaly, 224 Trikalon Street, GR 43100 Karditsa, Greece; vlachoumikvet@uth.gr (M.V.); nsolom@vet.uth.gr (N.S.); agovaris@vet.uth.gr (A.G.); 2 Department of Food Science and Nutrition, University of Thessaly, Terma N. Temponera Street, GR 43100 Karditsa, Greece; vaathanasiadis@uth.gr (V.A.); slalas@uth.gr (S.I.L.)

**Keywords:** ochratoxin A, pig tissues, pork meat, HPLC-FD, mycotoxins, Greece

## Abstract

This study aimed to assess the occurrence and contamination levels of OTA in edible tissues of slaughtered pigs in Greece using high-performance liquid chromatography with fluorescence detection (HPLC-FD). Kidney, liver, muscle, and fat samples were collected from 1695 healthy slaughtered pigs originating from 113 swine farms across eight geographical regions of Greece and analyzed for OTA. OTA was not detected in muscle or fat samples. In contrast, OTA was detected in 99 of 1695 kidney samples (5.8%), with concentrations ranging from 0.36 to 1.36 μg/kg (mean 0.73 μg/kg; median 0.70 μg/kg). OTA-positive kidney samples were identified in four regions, with the highest prevalence recorded in the regional unit of Karditsa, within the region of Thessaly (75/105 samples; 71.4%), where the maximum OTA concentration in kidneys was observed (1.36 μg/kg). Karditsa was also the only regional unit where OTA was detected in liver samples (40/1695; 2.4%), with concentrations ranging from 0.42 to 1.08 μg/kg (mean 0.61 μg/kg; median 0.53 μg/kg). The lack of detectable OTA levels in muscle and fat indicates minimal consumer exposure through pork; nevertheless, the presence of low-level residues in kidneys and liver emphasizes the necessity for ongoing monitoring using sensitive analytical methods. Overall, OTA contamination in edible tissues was low and unevenly distributed, reflecting localized exposure likely associated with region- or farm-specific feed contamination.

## 1. Introduction

Ochratoxin A (OTA), a mycotoxin produced mainly by *Aspergillus* and *Penicillium* species, is widely present in plant-derived commodities and animal feeds worldwide, allowing its incorporation into the food chain through both direct and indirect pathways [1]. OTA is of major toxicological concern due to its well-established nephrotoxic effects, as well as its immunotoxic, genotoxic, teratogenic, and carcinogenic properties [1,2]. In humans, chronic dietary exposure to OTA has been associated with nephrotoxic effects and the development of tumors in the urinary tract and is classified as a possible human carcinogen (Group 2B) by the International Agency for Research on Cancer (IARC) [3].

Among food-producing animals, pigs are considered particularly susceptible to OTA exposure due to the high bioavailability of the toxin and its slow elimination rate, which may favor residue formation in edible tissues following the consumption of contaminated feed [4,5]. OTA is widely present in feed materials and compound diets for pigs, resulting in measurable exposure, with contaminated feed representing the primary route of exposure [6]. After dietary exposure, OTA is systemically distributed, and a consistent tissue distribution pattern has been reported, with the highest concentrations detected in blood, followed by kidneys and liver, while lower levels are generally found in muscle and fat tissues. This distribution is highly relevant for food safety, as OTA residues in kidneys and liver, and, at lower levels, in muscle and fat tissues, may contribute to human exposure through pork and pork-derived products, particularly those containing organs or blood (from the transfer from feed to organs/tissues, “carry-over effect”). This concern has been highlighted in the EFSA risk assessment on OTA in food, while more recent EFSA evaluations underline the relevance of tissue residues in pigs, particularly in kidneys and liver, for assessing dietary exposure and potential residue formation [1,5].

Numerous studies have investigated the occurrence of OTA in edible pig tissues worldwide, reporting highly variable contamination levels ranging from non-detectable to relatively high concentrations. This variability is largely influenced by the type of tissue analyzed, as well as by geographic region, feeding practices, and the analytical methodologies employed. The highest OTA occurrence and concentrations are most frequently reported in blood, followed by kidneys and liver, whereas muscle and fat generally show lower prevalence and contamination levels [7,8,9,10,11,12,13]. This wide range of reported values highlights both the heterogeneous nature of OTA exposure in pigs and the critical importance of analytical sensitivity for accurately assessing residue occurrence in edible tissues. A variety of analytical techniques have been applied for the detection and quantification of OTA residues in pig tissues, with the most used methods including high-performance liquid chromatography with fluorescence detection (HPLC-FD), liquid chromatography–mass spectrometry (LC–MS), and enzyme-linked immunosorbent assay (ELISA) [13].

In Greece, available data on the occurrence of OTA in slaughtered pigs are limited and have mainly focused on blood serum. Previous large-scale studies conducted in Greece, employing ELISA as a screening method and supported by HPLC-FD, demonstrated a high prevalence of OTA in pig serum across multiple regions, indicating widespread exposure of animals to contaminated feed [14,15]. In contrast, OTA was not detected in kidneys, liver, muscle, or fat when analyzed by ELISA, likely due to both low contamination levels and the limited sensitivity of screening methods for these tissues [14]. Similar findings have been reported in other countries, where HPLC-based methods showed higher sensitivity and specificity for OTA determination in tissues, compared with immunochemical screening assays [16,17,18]. Moreover, in Greece, the sporadic detection of OTA in pig liver using the more sensitive LC-MS/MS approach [19] suggests that low-level residues in edible tissues may occur and remain undetected by screening techniques.

Against this background, the present study aims to investigate the occurrence and concentration of OTA in kidneys, liver, muscle, and fat of slaughtered pigs in Greece using HPLC-FD analysis, providing a more sensitive assessment of OTA residues in edible tissues and contributing to improved risk evaluation and monitoring strategies in the pork production chain.

## 2. Results

No OTA-positive samples were detected in muscle and fat tissues from slaughtered pigs in Greece. OTA occurrence was mainly observed in kidney samples, while liver contamination was limited and geographically restricted, occurring exclusively in the regional unit of Karditsa, within the region of Thessaly. Overall, OTA positivity in liver samples was very low, as shown in Table 1, with 40 of 1695 samples (2.4%) and 4 of 113 farms (3.5%) being OTA-positive, respectively. In the regional unit of Karditsa, 4 out of 7 farms (57.1%) and 40 out of 105 samples (38.1%) were positive, with OTA concentrations ranging from 0.42 to 1.08 μg/kg (mean: 0.61 μg/kg; median: 0.53 μg/kg). Accordingly, the following section focuses on the presence and regional distribution of OTA in pig kidneys across Greece.

### 2.1. Presence and Regional Distribution of OTA in the Kidneys of Slaughtered Pigs

The results on the occurrence and concentration levels of OTA in kidneys of slaughtered pigs in Greece, stratified by region and regional unit, are summarized in Table 2. Overall, OTA was detected in 99 out of 1695 kidney samples (5.8%), with concentrations ranging from 0.36 to 1.36 μg/kg. The mean OTA concentration was 0.73 μg/kg, with a median value of 0.70 μg/kg.

OTA-positive kidney samples were detected in 4 out of the 8 regions included in the study (Thessaly, Central Macedonia, Western Greece, and Crete). At the regional unit level, positivity was recorded in 4 out of the 21 units (Karditsa, Thessaloniki, Aetolia-Acarnania, and Heraklion). No OTA-positive kidney samples were detected in the remaining regions (Epirus, Central Greece, Eastern Macedonia and Thrace, and Peloponnese), nor in the other regional units sampled (see Table 2).

The highest OTA concentration (1.36 μg/kg) was detected in kidneys of slaughtered pigs from farms located in the region of Thessaly, specifically in the regional unit of Karditsa. In this regional unit, 2 out of 7 farms (28.6%) and 71.4% of the 105 kidney samples analyzed were OTA-positive, with concentrations ranging from 0.38 to 1.36 μg/kg (mean: 0.82 μg/kg; median: 0.79 μg/kg).

In Central Macedonia, OTA-positive kidney samples were detected in the regional unit of Thessaloniki, which exhibited the highest proportion of OTA-positive farms (3 out of 7; 42.9%), while only 8 of the 105 kidney samples (7.6%) were positive, with concentrations ranging from 0.36 to 0.56 μg/kg (mean: 0.47 μg/kg).

Similarly low positivity rates in kidney samples were observed in the region of Crete, specifically in the regional unit of Heraklion, where 5 out of 60 samples (8.3%) were OTA-positive, with concentrations ranging from 0.36 to 0.53 μg/kg. The mean and median OTA concentrations were 0.43 μg/kg and 0.42 μg/kg, respectively, while positive samples originated from only one of the four farms examined (25.0%). Comparable OTA concentration levels were observed in Western Greece, specifically in the Regional Unit of Aetolia-Acarnania, where 11 out of 165 kidney samples (6.7%) were OTA-positive. Only two out of 11 farms (18.2%) were OTA-positive, with concentrations ranging from 0.37 to 0.53 μg/kg (mean: 0.42 μg/kg; median: 0.40 μg/kg).

**Figure 1:**
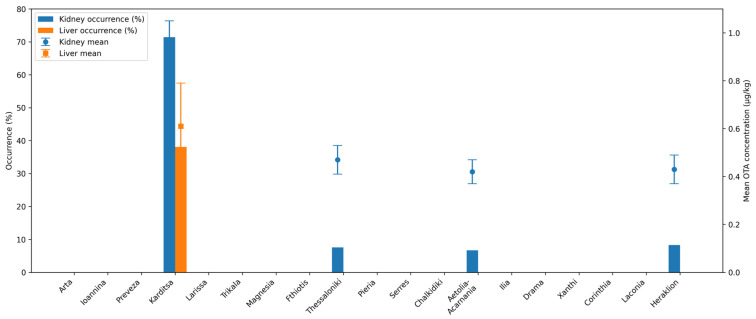
Occurrence (%) and mean concentration (μg/kg) of OTA in liver and kidneys of slaughtered pigs across regional units in Greece. Bars represent the percentage of positive samples, while dashed lines with markers indicate mean OTA concentrations. Error bars represent ± standard deviation (SD). Mean concentration and SD were calculated for positive samples only. Zero values indicate non-detectable levels.

The regional distribution of OTA occurrence and mean concentrations in pig tissues is illustrated in Figure 1, facilitating comparison among regional units and tissue types.

### 2.2. Prevalence of OTA Contamination in Kidneys on Farms by Region

The prevalence of OTA contamination in the kidneys of slaughtered pigs at the farm level was calculated as the number of OTA-positive samples among the 15 kidney samples examined per farm. Table 3 summarizes the farm-level prevalence of OTA contamination in the kidneys of slaughtered pigs across regions where OTA-positive samples were detected. In all regions, most farms were classified within the lowest prevalence range (0–25%), including Thessaly (77.3%), Central Macedonia (92.3%), Western Greece (93.3%), and Crete (75.0%). Higher prevalence levels (75–100%) were observed only in Thessaly (22.7%). Farms classified within the 25–50% prevalence range were identified in Central Macedonia (2/26; 7.7%) and Western Greece (1/15; 6.7%), with a higher proportion observed in Crete (1/4; 25.0%). No farms were classified within the 50–75% prevalence range.

## 3. Discussion

This study provides a nationwide HPLC-based analysis of edible tissues (kidney, liver, muscle and fat) from slaughtered pigs in Greece, showing OTA contamination predominantly in the kidneys, to a limited extent in the liver, and an absence of detectable residues in muscle or fat tissues. The higher OTA residues observed in kidneys and, to a lesser extent, in liver can be explained by the toxicokinetic behavior of the toxin in pigs. OTA exhibits a strong affinity for serum proteins, particularly albumin, resulting in prolonged circulation and preferential accumulation in highly perfused organs such as the kidneys and liver. The kidneys represent the primary target organ due to their role in toxin excretion, while the liver is also involved in OTA metabolism. This distribution pattern is consistent with previous studies on OTA tissue distribution in pigs [13].

OTA was detected in 5.8% of kidney samples, indicating a relatively low prevalence compared with that reported in several European countries, where detection rates range from 33% to nearly 100%, including Denmark, Italy, Germany, Romania, Serbia, France, and Belgium, based mainly on HPLC-FD and, more recently, LC-MS/MS analyses [7,12,20,21,22,23]. A prevalence comparable to that observed in the present study has been reported only in the Czech Republic (around 8%), based on HPLC-FD analysis [24]. In comparison, reported prevalences in non-European countries are generally lower, with OTA detected in pig kidneys in Canada at approximately 3–30% using HPLC-based methods [25], and in China at 0–10% when LC-MS/MS was applied [26].

In the present study, OTA concentrations in kidneys ranged from 0.36 to 1.36 μg/kg, which is consistent with concentration ranges reported in several European countries, including France (0.40–1.40 μg/kg) [20], Italy (0.26–3.05 μg/kg) [23], and the Czech Republic (0.15–0.46 μg/kg) [24]. Comparable OTA concentrations in pig kidneys have also been reported in non-European countries, including China (0.03–0.32 μg/kg) [27] and Canada, where OTA levels in pig kidneys were generally reported at or below approximately 1 μg/kg [25].

The wide variability in OTA prevalence in pigs’ kidneys reported among studies, considering differences in sampling design and analytical methodology, reflects variability in animal exposure levels and duration as well as temporal and regional fluctuations in feed contamination [12,27]. Differences in OTA contamination levels have been reported in feeds among countries and are associated with differences in feed production systems, including conventional and ecological farming practices, feed composition, feed storage conditions, and environmental and climatic factors [10,18,28,29,30,31,32].

A pronounced regional heterogeneity was observed within Greece, with the highest prevalence and concentrations recorded in the regional unit of Karditsa, within the region of Thessaly, where 71.4% of kidney samples were OTA-positive and the maximum concentration reached 1.36 μg/kg. In contrast, other OTA-positive regions (Central Macedonia, Western Greece, and Crete) showed much lower prevalence (6.7–8.3%) and narrower concentration ranges (≤0.56 μg/kg).

A plausible explanation for the markedly higher OTA prevalence observed in the regional unit of Karditsa may relate to region-specific factors affecting feed contamination. Thessaly is characterized by intensive cereal production, particularly maize, which is widely used in swine diets and is known to be highly susceptible to fungal growth and OTA formation under suboptimal storage conditions. Moreover, the region frequently experiences periods of elevated humidity and temperature fluctuations, conditions that favor the proliferation of *Aspergillus* and *Penicillium* species in stored feed. In addition, many pig farms in this area operate with on-farm storage of cereals, where variability in storage infrastructure, aeration, and moisture control may contribute to heterogeneous contamination patterns. Although feed samples were not analyzed in the present study, these factors collectively support the hypothesis that localized feed contamination is the primary driver of the high OTA burden observed in pigs from Karditsa. This interpretation aligns with previous reports indicating that OTA occurrence in pig tissues often reflects farm- or region-specific feed management practices rather than uniform national exposure.

Based on the present results, OTA contamination in pig kidneys at the farm level was generally low across regions, with most farms classified within the lowest prevalence range (0–25%). Nevertheless, the occurrence of farms with high prevalence in the region of Thessaly, particularly in the regional unit of Karditsa, indicates a non-uniform distribution of OTA exposure at the farm level. A similar farm-to-farm variability has been reported by Polovinski-Horvatovic et al. [12], who observed differences in OTA incidence among pig farms in Serbia, including farms with only sporadic positive samples. Such farm-specific differences have been attributed to variations in the timing and magnitude of OTA contamination in feed, which may influence the proportion of pigs testing positive within individual farms.

Also, OTA contamination in liver was geographically restricted to the regional unit of Karditsa, which was the only area with OTA-positive liver samples, showing a prevalence of 12.1%, while the total prevalence across all regions was markedly lower (2.4%). The total prevalence of OTA in liver samples from slaughtered pigs in Greece is substantially lower than that reported in most European studies using chromatographic methods, where positivity rates ranged from 17% to 100% [7,21,33,34]. Nevertheless, the concentration range detected in liver samples in the present study (0.42–1.08 μg/kg) is comparable to values reported in several European countries, including Denmark, Romania, and Italy [7,33,34]. Substantially higher concentrations have been reported in Italy (3.2–5.3 μg/kg) [35] and in Serbia (up to 14.5 μg/kg) [36]. In non-European countries, OTA occurrence in pig livers has also been reported, although generally at lower prevalence levels. In China, OTA was detected in approximately 33% of pig liver samples, with reported concentrations around 1.46 μg/kg [37]. In Canada, OTA was sporadically detected in pig livers, with concentrations generally reported in the low μg/kg range (≤1 μg/kg), comparable to those observed in the present study [25].

Overall, the regional heterogeneity observed across different regions of Greece with respect to OTA contamination of pig kidney and liver aligns with findings reported worldwide, where OTA occurrence is associated with localized feed contamination rather than uniform national exposure [12,13,27,36,38]. This is further supported by previous findings from Greece, where OTA was detected in liver samples from only one out of eight pig farms sampled across different regions of the country, indicating a similarly localized and farm-specific occurrence [19]. These observations highlight the importance of region-specific monitoring strategies for both animal tissues and feed.

Taken together, the international evidence and the present findings indicate that OTA contamination in pigs is characterized by substantial heterogeneity, both between and within countries. This variability reflects differences in feeding practices, storage conditions, climatic factors, and the analytical methods applied across studies. In this context, the results from Greece fit well within the broader European pattern, where OTA occurrence in kidneys and liver is generally low but can exhibit localized peaks linked to region-specific feed contamination. This broader perspective provides an essential framework for interpreting the tissue-specific findings of the present study, particularly regarding the absence of detectable OTA in muscle and fat.

In Greece, previous investigations on OTA in slaughtered pigs have primarily focused on serum, with ELISA used as the main screening tool. These studies consistently reported high OTA prevalence in blood but failed to detect the toxin in edible tissues such as kidneys, liver, muscle, or fat. This discrepancy is likely attributable to the limited analytical sensitivity of ELISA for complex tissue matrices, where OTA concentrations are typically low and often close to the detection limits of immunochemical assays. The sporadic detection of OTA in pig liver using LC-MS/MS in a recent Greek study further supports the presence of low-level residues that may remain undetected when less sensitive methods are applied. By employing HPLC-FD with validated LOD and LOQ values suitable for tissue analysis, the present study provides the first large-scale, analytically robust evidence of OTA occurrence in edible pig tissues in Greece. These findings highlight the importance of using chromatographic methods for accurate residue assessment and demonstrate that previous reports of non-detectable OTA levels in tissues likely reflected methodological limitations rather than true absence of contamination.

No OTA was detected in muscle or fat tissues in the present study, consistent with reports describing either non-detectable or very low OTA levels in pork meat and adipose tissue under conditions of limited overall exposure. For example, Wei et al. [26] reported no detectable OTA in muscle and fat samples from pigs in China using LC-MS/MS, while Škarková et al. [24], using HPLC-FD, detected OTA in only 8% of muscle samples in the Czech Republic, with concentrations close to the analytical detection limit (LOD 0.10 μg/kg). Nevertheless, OTA presence in muscle tissue has been reported in several studies, particularly when highly sensitive analytical methods were applied or exposure levels were elevated. In Germany, OTA was detected in 7.7–17.2% of muscle samples using HPLC-FD [21], whereas in Denmark, Jørgensen and Petersen [22] reported positivity rates up to 76%, although concentrations were generally low. Similarly, in France, Hort et al. [11] reported positivity rates of 67–76% in muscle samples analysed by SIDA–UHPLC–MS/MS, particularly in pigs from organic production systems. Despite these relatively high detection frequencies, OTA concentrations in muscle were typically low, most often ranging from trace levels to ≤0.15 μg/kg and were frequently close to the analytical detection or quantification limits. In Italy, Meucci et al. [39] reported OTA concentrations of only 0.079–0.085 μg/kg in pig fat using HPLC-FD, regardless of the production system. Higher OTA levels in fat have been rarely described; an exception is the study by Pleadin et al. [10] in Croatia, which reported concentrations of 2.95–5.26 μg/kg.

The absence of detectable OTA in muscle and fat in the present study should be interpreted in light of both toxicokinetic and analytical considerations. This toxicokinetic profile explains the preferential accumulation of OTA in kidneys and liver and its limited presence in muscle and fat tissues. Consequently, even under conditions of moderate exposure, OTA concentrations in muscle and fat are expected to remain very low and often close to the analytical detection limits of chromatographic methods. Although the HPLC-FD method applied in this study provides adequate sensitivity for tissue analysis, it is possible that trace-level residues below the LOQ may remain undetected, particularly in muscle, where OTA concentrations reported internationally frequently fall within the sub-LOQ range. Therefore, the non-detectable levels observed here are consistent with the known toxicokinetic profile of OTA in pigs and with previous studies reporting minimal carry-over into muscle and fat under low-exposure scenarios.

From a public health perspective, the low OTA concentrations detected in kidneys and liver, combined with the absence of detectable residues in muscle and fat, indicate that consumer exposure through pork is expected to be minimal under current conditions. According to EFSA, the contribution of animal-derived foods to total dietary OTA intake in Europe is generally low, with plant-based commodities representing the primary exposure sources. Even in the regional unit of Karditsa, where the highest tissue concentrations were observed, the detected levels remain far below those associated with acute toxicity and are unlikely to significantly influence overall dietary exposure. Nevertheless, the presence of OTA in edible organs is not irrelevant, particularly for consumers who frequently consume offal or traditional products incorporating liver or kidney. Therefore, continued monitoring of OTA in both animal tissues and feed is essential to ensure that localized contamination events do not lead to increased exposure in specific population subgroups.

The findings of this study highlight the importance of maintaining targeted monitoring programs for OTA in both animal tissues and feed, despite the generally low contamination levels observed. Currently, no maximum residue limits (MRLs) for OTA in pig tissues are established within the European Union, and regulatory focus is primarily directed toward OTA in plant-derived food products, as defined in Commission Regulation (EU) 2023/915 [40], but not in foods of animal origin. Although some countries have established national limits for OTA in pig tissues, no such limits exist in Greece, where legislation is harmonized under the EU framework [13]. Importantly, the OTA concentrations detected in kidneys and liver in this study were well below levels considered to pose a toxicological concern, and therefore do not indicate any immediate risk for consumers. The detection of OTA in kidneys and liver in specific regions of Greece, particularly in Thessaly, underscores the need for region-specific surveillance strategies and reinforces the relevance of routine feed testing as a preventive measure. Although the concentrations detected in this study do not raise immediate food safety concerns, the localized nature of contamination suggests that regulatory authorities should continue to prioritize good agricultural and storage practices, implement risk-based sampling plans, and ensure that farms in high-risk areas receive appropriate guidance on feed management. Strengthening these preventive measures would help minimize the likelihood of future contamination events and support the long-term safety of the pork production chain.

Taken together, the present findings place earlier nationwide Greek tissue surveys in context, indicating that the absence of detectable OTA residues when ELISA was applied reflects methodological sensitivity rather than the complete absence of tissue exposure [14]. The identification of OTA in kidneys and liver by HPLC-based analysis demonstrates that low-level residues may occur under chronic exposure conditions but remain below the detection capability of screening assays. This interpretation is consistent with comparative serum data showing substantially higher detection rates with HPLC-FD than with ELISA [15], as well as with reports highlighting the superior sensitivity of chromatographic techniques for OTA determination in animal tissues [16,17,18].

In the present study, the observed tissue distribution pattern (kidney > liver > muscle-fat) is consistent with experimental and field studies and reflects the well-established toxicokinetic behavior of OTA in pigs, with renal tissue representing the primary site of accumulation, followed by the liver, while substantially lower residues are typically detected in muscle and fat tissues [7,13,14,41,42]. Differences in OTA occurrence and concentrations among studies have been primarily associated with variability in exposure dose and duration, the nature of contamination (natural versus experimental), the time interval between exposure and slaughter and the analytical method employed [13,28].

Despite the insights provided, some limitations should be acknowledged. The use of HPLC-FD, although suitable for OTA determination, is less sensitive than advanced techniques such as UPLC-MS/MS, and trace-level residues may have remained undetected. In addition, OTA metabolites, such as ochratoxin α (OTα), were not analyzed, which may lead to an underestimation of total toxin burden.

## 4. Conclusions

In conclusion, this nationwide investigation provides the most comprehensive and analytically robust assessment to date of OTA occurrence in edible pig tissues in Greece. OTA contamination was predominantly detected in kidneys and, to a lesser extent, in liver, while muscle and fat consistently tested negative. Although overall contamination levels were low, the pronounced regional clustering observed, particularly in the regional unit of Karditsa, highlights the influence of localized feed contamination and underscores the importance of region-specific monitoring strategies. The findings confirm that consumer exposure to OTA through pork is expected to be minimal under current conditions; however, the detection of OTA in edible organs reinforces the need for continued surveillance of both animal tissues and feed materials. Strengthening preventive measures in feed storage and management, especially in high-risk areas, will be essential to further reduce the likelihood of contamination events. Overall, the study contributes valuable data to national and European risk assessment efforts and supports the ongoing development of targeted monitoring programs aimed at safeguarding food safety within the pork production chain.

## 5. Materials and Methods

### 5.1. Sample Collection

**Figure 2:**
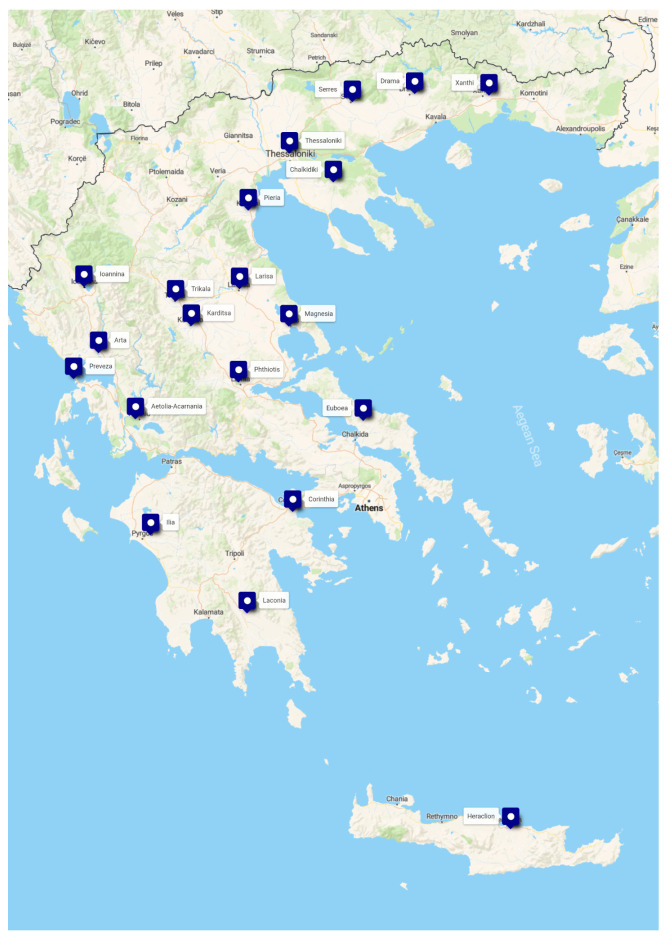
Geographical distribution of sampling locations from pig farms and slaughtered pigs in Greece.

During the period from November 2018 to April 2021, a total of 1695 randomly selected healthy slaughtered pigs were included in the survey. Sampling was conducted at slaughterhouses, and the pigs originated from 113 swine farms (15 pigs per farm) located in 21 regional units across eight geographical regions of Greece (Epirus, Thessaly, Western Macedonia, Central Greece, Central Macedonia, Eastern Macedonia and Thrace, Peloponnese, and Crete). The geographical distribution of sampling locations is shown in Figure 2. The distribution of sampled farms and collected samples by region and regional unit is presented in Table 4. These regions represent areas with significant swine production, according to data from the Ministry of Rural Development and Food of Greece (2017).

Slaughtering was performed in licensed slaughterhouses under official veterinary supervision, in full compliance with Council Regulation (EC) No 1099/2009 on the protection of animals at the time of killing [43]. Post-mortem inspection of all slaughtered pigs revealed no macroscopic kidney lesions or other pathological findings indicative of nephropathy or systemic disease. Following slaughter, tissue samples were collected from each pig carcass, including more than 100 g of liver, muscle and fat tissue, and one whole kidney. All samples were stored at −20 °C until OTA analysis. No preservatives were added, and samples were thawed immediately prior to analysis, as described below.

### 5.2. HPLC-FD Analysis

#### 5.2.1. Sample Preparation

Pig tissue samples (kidney, liver, muscle, and adipose tissue) were analyzed for ochratoxin A (OTA) according to a protocol adapted from Pleadin et al. [10], including extraction, clean-up, and concentration steps. All chemicals and solvents used were of analytical or HPLC grade. For extraction, 5 g of homogenized tissue was mixed with 7.5 mL of 1% aqueous sodium hydrogen carbonate (NaHCO_3_) solution (Chem-Lab NV, Zedelgem, Belgium) and stirred for 5 min. Subsequently, 17.5 mL of methanol (Chem-Lab NV, Zedelgem, Belgium) was added, and the mixture was vortexed for 1 min, followed by homogenization for 30 min. The samples were then centrifuged at 3500× *g* for 10 min at room temperature. A defatting step was performed by adding 10 mL of hexane (Scharlab, Barcelona, Spain), shaking for 3 min, and discarding the upper organic phase; this step was repeated once. Then, 0.25 mL of 0.4 mol/L silver nitrate (AgNO_3_) solution (Alfa Aesar GmbH & Co. KG, Karlsruhe, Germany) was added to 5 mL of the extract, followed by centrifugation under the same conditions.

The resulting supernatant was purified and concentrated using immunoaffinity columns (OCHRAPREP^®^, Product Code: P14/P14B, R-Biopharm AG, Darmstadt, Germany) at an approximate flow rate of one drop per second. Prior to sample loading, the column was conditioned with 1 mL of phosphate-buffered saline (PBS). A 5 mL aliquot of the sample extract was then applied, and the sample container was rinsed with 5 mL of PBS (pH 7.4), which was also passed through the column. The PBS solution was prepared by dissolving NaCl (8.0 g), Na_2_HPO_4_ (1.16 g), KH_2_PO_4_ (0.2 g), and KCl (0.2 g) (all from Chem-Lab NV, Zedelgem, Belgium) in 1 L of deionized water obtained from a Milli-Q system (Millipore, Milford, CT, USA). The column was washed with 10 mL PBS and allowed to dry for 30 s. OTA was eluted using 1.5 mL of methanol/acetic acid (98:2, *v*/*v*) (Chem-Lab NV, Zedelgem, Belgium), passed through the column three times. The eluate was diluted with 1.5 mL of deionized water and stored at −18 °C until HPLC-FD analysis.

#### 5.2.2. Determination of OTA by HPLC-FD

OTA detection and quantification were performed using HPLC-FD, following the method described by Pleadin et al. [10]. Analyses were carried out using a Shimadzu HPLC system comprising a system controller (CBM-20A), an autosampler (SIL-20AC), a column oven (CTO-20AC), and a fluorescence detector (RF-10AXL) (Shimadzu Europa GmbH, Duisburg, Germany). Fluorescence detection was set at 334 nm (excitation) and 460 nm (emission).

Chromatographic separation was achieved on a C18 column (Phenomenex Luna C18(2), 250 × 4.6 mm, 5 μm; Phenomenex Inc., Torrance, CA, USA) under isocratic conditions using a mobile phase of acetonitrile/water/isopropanol/acetic acid (46/46/6/2, *v*/*v*/*v*/*v*) at a flow rate of 1 mL/min. The column temperature was maintained at 40 °C, and the injection volume was 100 μL. The retention time of OTA was 18.4 ± 0.2 min.

**Figure 3:**
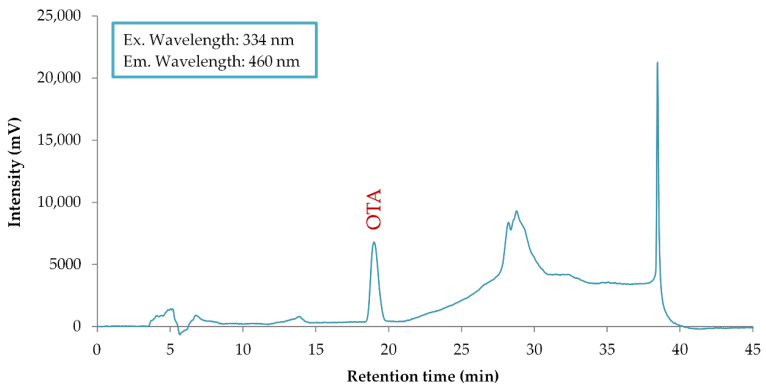
Representative HPLC-FD chromatogram obtained from an OTA standard solution (1.0 μg/L).

Quantification was performed by external calibration using OTA standard solutions in the concentration range of 0–1.00 μg/L, prepared by serial dilution of a certified standard (Trilogy^®^ Liquid Standard Ochratoxin A, R-Biopharm AG). The lowest calibration level (minimum OTA standard) was 0.05 μg/L, which ensured adequate sensitivity and reliable quantification near the lower concentration range. A representative HPLC-FD chromatogram of the OTA standard solution (1.0 μg/L) is shown in Figure 3.

The limits of detection (LOD) and quantification (LOQ) were initially estimated based on the methodology described by Pleadin et al. [10]; however, both parameters were experimentally verified and adjusted under the present laboratory conditions to ensure method suitability for the specific tissue matrices analyzed. Specifically, LOD and LOQ values were determined through repeated analysis of tissue samples spiked at low OTA concentrations, following standard analytical validation criteria (signal-to-noise ratios of 3:1 and 10:1, respectively).

The resulting LOD and LOQ values were as follows: kidney (0.24 μg/kg and 0.36 μg/kg), liver (0.36 μg/kg and 0.42 μg/kg), muscle (0.16 μg/kg and 0.22 μg/kg), and fat (0.23 μg/kg and 0.29 μg/kg).

Method performance was further evaluated through recovery and reproducibility studies. Accuracy (recovery) was assessed using spiked samples at multiple concentration levels, while precision (repeatability) was evaluated through intra-day and inter-day analyses. Instrumental stability and method precision were assessed by periodic injection of OTA standard solutions, including the injection of a 0.10 μg/L OTA standard after every five injections, and analysis of spiked samples at different concentration levels, using established quality control procedures. Overall, the method demonstrated satisfactory linearity (R^2^ > 0.99) within the studied concentration range, along with acceptable accuracy, precision, and negligible matrix effects, confirming its robustness and suitability for the determination of OTA in pig tissues.

### 5.3. Statistical Analysis

Statistical analysis of HPLC-FD data [mean, median, and coefficient of variation (CV)] was performed using Statistica version 6.1 (StatSoft Inc., Tulsa, OK, USA). Statistical significance was set at *p* = 0.05. Only positive samples (>LOD) were included in the analysis. A farm was classified as positive when at least one OTA-contaminated sample was detected. Values between LOD and LOQ were substituted with ½ LOQ [44].

## Figures and Tables

**Table 1:** Occurrence and concentration levels of OTA in livers of slaughtered pigs in Greece.

Region	Regional Unit	Farms (*N*)	Positive Farms *n* (%)	Samples (*N*)	Positive Samples *n* (%)	Mean * (μg/kg)	SD *	Median * (μg/kg)	CV *	Range (μg/kg)
Thessaly	Karditsa	7	4 (57.1)	105	40 (38.1)	0.61	0.18	0.53	0.29	0.42–1.08
	Total	22	4 (18.2)	330	40 (12.1)	0.61	0.18	0.53	0.29	0.42–1.08
Whole Total		113	4 (3.5)	1695	40 (2.4)	0.61	0.18	0.53	0.29	0.42–1.08

* Statistical analysis included only positive samples. CV = coefficient of variation; *N* = total number of samples examined; *n* = number of positive samples (see Section 2).

**Table 2:** Occurrence and concentration levels of OTA in kidneys of slaughtered pigs in Greece by region.

Region	Regional Unit	Farms (*N*)	Positive Farms *n* (%)	Samples (*N*)	Positive Samples *n* (%)	Mean * (μg/kg)	SD *	Median * (μg/kg)	CV *	Range (μg/kg)
Thessaly	Karditsa	7	2 (28.6)	105	75 (71.4)	0.82	0.23	0.79	0.28	0.38–1.36
	Total	22	2 (9.1)	330	75 (22.7)	0.82	0.23	0.79	0.28	0.38–1.36
Central Macedonia	Thessaloniki	7	3 (42.9)	105	8 (7.6)	0.47	0.06	0.47	0.14	0.36–0.56
	Total	26	3 (11.5)	390	8 (2.05)	0.47	0.06	0.47	0.14	0.36–0.56
Western Greece	Aetolia-Acarnania	11	2 (18.2)	165	11 (6.7)	0.42	0.05	0.40	0.13	0.37–0.53
	Total	15	2 (13.3)	225	11 (4.9)	0.42	0.05	0.40	0.13	0.37–0.53
Crete	Heraklion	4	1 (25.0)	60	5 (8.3)	0.43	0.06	0.42	0.15	0.36–0.53
	Total	4	1 (25.0)	60	5 (8.3)	0.43	0.06	0.42	0.15	0.36–0.53
Whole Total		113	8 (7.1)	1695	99 (5.8)	0.73	0.26	0.70	0.36	0.36–1.36

* Statistical analysis included only positive samples. CV = coefficient of variation; *N* = total number of samples examined; *n* = number of positive samples (see Section 2).

**Table 3:** Prevalence of OTA contamination in kidneys of slaughtered pigs on farms by region in Greece.

Region	Prevalence Rate (Range)			
	0–25% *n* (%)	25–50% *n* (%)	50–75% *n* (%)	75–100% *n* (%)
Thessaly (*N* = 22)	17 (77.3)	0 (0.0)	0 (0.0)	5 (22.7)
Central Macedonia (*N* = 26)	24 (92.3)	2 (7.7)	0 (0.0)	0 (0.0)
Western Greece (*N* = 15)	14 (93.3)	1 (6.7)	0 (0.0)	0 (0.0)
Crete (*N* = 4)	3 (75.0)	1 (25.0)	0 (0.0)	0 (0.0)

*N* = total number of tested farms; *n* = number of farms.

**Table 4:** Distribution of sampled farms and collected samples from slaughtered pigs by region and regional unit in Greece.

Region	Regional Unit	Farms (*N*)	Samples (Kidney, Liver, Muscle and Fat) (*N*)
Εpirus	Arta	3	45
	Ioannina	5	75
	Preveza	9	135
	Total	17	255
Τhessaly	Karditsa	7	105
	Larissa	7	105
	Trikala	7	105
	Magnesia	1	15
	Total	22	330
Central Greece	Euboea	15	225
	Phthiotis	4	60
	Total	19	285
Central Macedonia	Thessaloniki	7	105
	Pieria	11	165
	Serres	6	90
	Chalkidiki	2	30
	Total	26	390
Western Greece	Aetolia-Acarnania	11	165
	Ilia	4	60
	Total	15	225
Eastern Macedonia and Thrace	Drama	3	45
	Xanthi	2	30
	Total	5	75
Peloponnese	Corinthia	2	30
	Laconia	3	45
	Total	5	75
Crete	Heraklion	4	60
	Total	4	60
Whole Total		113	1695

## Data Availability

The original contributions presented in this study are included in the article. Further inquiries can be directed to the corresponding author.

## Abbreviations

The following abbreviations are used in this manuscript:

OTA	Ochratoxin A
HPLC-FD	High-Performance Liquid Chromatography with Fluorescence Detection
LC–MS/MS	Liquid Chromatography–Tandem Mass Spectrometry
ELISA	Enzyme-Linked Immunosorbent Assay
LOD	Limit of Detection
LOQ	Limit of Quantification
CV	Coefficient of Variation
PBS	Phosphate-Buffered Saline
NaHCO_3_	Sodium Hydrogen Carbonate
AgNO_3_	Silver Nitrate
EFSA	European Food Safety Authority
IARC	International Agency for Research on Cancer
MRLs	Maximum Residue Limits
